# SUPPORTING CARE PARTNERS OF PEOPLE LIVING WITH DEMENTIA: EXPLORING CHANGES IN POLICY AND PRACTICE

**DOI:** 10.1093/geroni/igad104.2889

**Published:** 2023-12-21

**Authors:** Walter Dawson, Jenn Hollandsworth Reed, Anaeliz Colon, Devlin Prince, Sofia Chapela, Sherril Gelmon

**Affiliations:** OHSU-PSU School of Public Health, Portland, Oregon, United States; OHSU-PSU School of Public Health, Portland, Oregon, United States; OHSU-PSU School of Public Health, Portland, Oregon, United States; OHSU-PSU School of Public Health, Portland, Oregon, United States; OHSU-PSU School of Public Health, Portland, Oregon, United States; OHSU-PSU School of Public Health, Portland, Oregon, United States

## Abstract

Most care and support for people living with Alzheimer’s Disease and related dementias (ADRD) is provided unpaid at home or in community settings. To better understand the caregiving-related priorities of people with lived experience as care partners (CP) of people living with ADRD, focus groups were convened with CPs who identify with historically and currently underserved communities that may have difficulty accessing culturally specific services for people living with ADRD in and near Portland, Oregon. In-depth interviews (n=20) were conducted with local, state, and national-level organizational leaders who represent or provide ADRD support services to various underserved communities including Black, Indigenous, Asian, and Latinx Americans to gather additional perspectives on ADRD care and support needs. Care partners across all populations reported a need for additional supports that could help them in carrying out their caregiving responsibilities. While the specific needs and types of supports identified varied across populations, the findings point to multiple unmet needs across all populations in terms of ADRD CP-specific supports. Changes to policy and practice are needed to improve the experience of CPs of people living with ADRD. New policies focused on promoting access to services, education and training, and financing of supports such as respite must be developed and implemented in partnership with community-based organizations and ADRD CPs from underserved communities and communities that may have difficulty accessing culturally-specific services and unique supports. The findings also point to universal needs related to caregiving across all populations that could be addressed through new policy initiatives.

